# Clinical Applications of Polyetheretherketone in Removable Dental Prostheses: Accuracy, Characteristics, and Performance

**DOI:** 10.3390/polym14214615

**Published:** 2022-10-31

**Authors:** Yuchen Liu, Ming Fang, Ruifeng Zhao, Hengyan Liu, Kangjie Li, Min Tian, Lina Niu, Rui Xie, Shizhu Bai

**Affiliations:** 1 State Key Laboratory of Military Stomatology, School of Stomatology, The Fourth Military Medical University, Xi’an 710032, China; 2 National Clinical Research Center for Oral Diseases, School of Stomatology, The Fourth Military Medical University, Xi’an 710032, China; 3 Shaanxi Key Laboratory of Stomatology, School of Stomatology, The Fourth Military Medical University, Xi’an 710032, China; 4 Department of Prosthodontics, School of Stomatology, The Fourth Military Medical University, Xi’an 710032, China; 5 Digital Dentistry Center, School of Stomatology, The Fourth Military Medical University, Xi’an 710032, China

**Keywords:** accuracy, clinical performance, polyetheretherketone, removable prostheses

## Abstract

The high-performance thermoplastic polyetheretherketone (PEEK) has excellent mechanical properties, biocompatibility, chemical stability, and radiolucency. The present article comprehensively reviews various applications of PEEK in removable dental prostheses, including in removable partial dentures (RPDs) (frameworks and clasps), double-crown RPDs, and obturators. The clinical performance of PEEK in removable dental prostheses is shown to be satisfactory and promising based on the short-term clinical evidence and technical complications are scarce. Moreover, the accuracy of RPDs is a vital factor for their long-term success rate. PEEK in removable dental prostheses is fabricated using the conventional lost-wax technique and CAD/CAM milling, which produces a good fit. Furthermore, fused deposition modeling is considered to be one of the most practical additive techniques. PEEK in removable prostheses produced by this technique exhibits good results in terms of the framework fit. However, in light of the paucity of evidence regarding other additive techniques, these manufacturers cannot yet be endorsed. Surface roughness, bacterial retention, color stability, and wear resistance should also be considered when attempting to increase the survival rates of PEEK removable prostheses. In addition, pastes represent an effective method for PEEK polishing to obtain a reduced surface roughness, which facilitates lower bacterial retention. As compared to other composite materials, PEEK is less likely to become discolored or deteriorate due to wear abrasion.

## 1. Introduction

As a result of their affordability and conservatism, removable dental prostheses are utilized for temporary and long-term oral rehabilitation in fully and partially edentulous people [1]. Currently, metal alloys such as cobalt–chromium (CoCr) and titanium are appropriate and compatible with manufacturers of removable dental prostheses due to their high strength and stiffness, corrosion resistance, and repassivation properties [2]. However, the use of metal alloys causes esthetic issues, the potential risk of metallic flavor, and allergies. Moreover, metal ions and particles have the potential to cause cytotoxicity, hypertension, and neoplasia [3].

The rapid advancement of materials and the rapid evolution of computer-aided design and computer-aided manufacturing (CAD/CAM) technology have given rise to polyetheretherketone (PEEK), a high-performance thermoplastic semi-crystalline polymer. PEEK is a promising alternative material for metal-free removable dental prostheses that presents favorable characteristics such as superior mechanical properties, good thermal and chemical resistance, and radiographic radiolucency. Moreover, it can be used to avoid the silver color, which is unpopular, and allergies that are commonly observed in conventional metal prostheses [2]. Moreover, the low elastic modulus of PEEK warrants lighter removable dental prostheses and provides a cushioning effect on occlusal forces. Given these positive attributes, PEEK-based removable dental prostheses were studied to overcome the drawbacks of conventional prosthodontic metal materials [1]. 

PEEK can be obtained via the chemical reaction between 4,40-difluorobenzophenone and the disodium salt of hydroquinone in a polar solvent such as diphenyl sulphone. It is a semicrystalline material with a melting point between 370 and 400 °C depending on the polymer’s molar mass and the type and processing conditions used in manufacturing [3,4,5]. There are two main methods of processing PEEK in removable prostheses. On the one hand, a prosthesis can be milled from CAD/CAM blocks, and on the other hand, vacuum heat-pressing (from granules or pellets) can be used [4,5]. Recently, 3D printing has been used for the fabrication of removable PEEK prostheses. This type of additive manufacturing results in more accurate end products and provides the potential to fabricate complex designs [6]. Removable PEEK prostheses must fit adequately. A recent systematic review concluded that PEEK RPDs produced by rapid prototyping exhibited good results in terms of the framework fit [7]. However, evidence is needed that can determine the accuracy and precision of RPDs made of PEEK in comparison with other metal materials and help select the optimum manufacturing method.

Some characteristics are closely related to the long-term use of removable dental prostheses, such as surface roughness, bacterial retention, color stability, and wear properties. Reduced surface roughness plays a significant function in improving tongue sensation and in avoiding adherence to dental plaque. The previous research suggested that PEEK prostheses needed proper polishing protocols before clinical applications [8]. However, the most effective polishing protocols for PEEK remain to be established. In terms of bacterial retention, PEEK differs from other materials [9], as summarized in the latter part of this review. Color stability and wear resistance are both vital clinical characteristics for dental restorations. However, the authors are unaware of a previous review that has summarized the data on these characteristics.

Therefore, the objective of this review is to evaluate the accuracy of removable prostheses made of PEEK in comparison with other metal materials and help select the optimum manufacturing method. Moreover, the long-term use of PEEK removable prostheses and the recent literature data on the characteristics of PEEK and its composite materials, such as surface roughness, bacterial retention, color stability, and PEEKs wear properties, are summarized. Finally, the clinical performance of different PEEK removable dental prostheses was evaluated based on currently available evidence.

## 2. Accuracy of CAD/CAM Fabrication for PEEK Materials

PEEK is widely used to fabricate removable restorations, which are often manufactured using the conventional lost-wax technique, via heat-pressing pellets or granules, or using CAD/CAM techniques, which can be classified as subtractive or additive (3D printing) techniques [7,10]. 

The suitability and accuracy of removable prostheses are important and key points that can be used to evaluate their clinical performance. Good suitability facilitates maximum support, retention, and stability in removable prostheses [11], and it helps avoid adverse mechanical stimulation and the negative effects caused by biological factors during masticatory force [12]. An inadequate fit may accelerate abutment movement and lower masticatory efficiency, with poor retention being the chief problem during functional movement [13]. In terms of the accuracy and fit of the frameworks fabricated using different techniques, various methods are used, such as the “impression method” [12], “light microscopy method” [14], and “digital methods using 3D software” [11,15]. For removable prosthesis frameworks, there is a wider tolerance range for the level of accuracy that is considered clinically acceptable as compared to fixed prostheses. This is due to the elastic residual alveolar ridge, which can be deformed when the prosthesis sinks under masticatory force [13]. A gap thickness from 0 to 50 μm between frameworks and casts is regarded as close contact (no gap) [12,13,16], and a gap thickness from 50 to 311 μm is considered to be a clinically acceptable fit [16].

Various studies have compared the accuracy of conventional materials (CoCr) and PEEK. In some clinical practices [17,18], PEEK has emerged as an esthetic alternative to removable prostheses for CoCr, as the accuracy of these removable prosthesis frameworks has exhibited a good fit [7]. A dental technique study reported that the average trueness of removable partial dentures with a PEEK framework was 0.03 mm [17], which is in accordance with a laboratory study [15] that qualitatively and quantitatively evaluated the in vitro fit of frameworks milled from PEEK. Qualitative assessments included a visual inspection and a pressing test. Gaps were duplicated using a silicone impression material between the removable partial dentures, and 3D digital software was used to take quantitative thickness measurements of the casts. For the entirety and all areas (occlusal rests, major connector, denture base) of the gap thickness, the frameworks milled from PEEK exhibited a more superior fit as compared to traditional cast frameworks. This is in line with a study by Arnold et al. [14]. This study evaluated the fit of removable partial denture clasps produced using four different CAD/CAM systems and the conventional lost-wax casting technique. The milled-PEEK clasps exhibited a markedly better fit in both the vertical and horizontal directions than the CoCr fabricated using the lost-wax casting technique and CAD/CAM milling. For other materials, Guo et al. [19] compared the spaces between the tissue surfaces of removable space maintainers (RSMs) and cast models. The results from this study found that PEEK RSMs fit the models well compared to conventional RSMs fabricated using polymer powder. However, further clinical and laboratory studies are needed to evaluate the accuracy of removable PEEK prostheses.

In terms of the accuracy of different fabrication techniques, Negm et al. [11] compared the accuracy of maxillary removable partial denture frameworks made of PEEK and fabricated by direct (milled by PEEK blocks) and indirect CAD/CAM techniques (resin 3D printing combined with the heat-press technique). Metrology software was used to measure misfits at 25 standardized points. The PEEK frameworks fabricated by the indirect technique had lower overall trueness values (71.68 ± 17.54 mm) than those fabricated using direct techniques (17.36 ± 4.32 mm). Conversely, an in vitro study by Lalama [20] evaluated the accuracy of PEEK (with 20% ceramic fillers)-based post and core restorations fabricated using the conventional lost-wax technique and milling systems and showed that the conventional PEEK posts and cores had a significantly higher accuracy than the posts and cores made from milled PEEK (*p* = 0.0047). The authors determined which fillers could have a negative effect on CAD/CAM-fabricated posts and cores. The accuracy of fabrication techniques under different clinical applications may vary.

Additive PEEK techniques mainly focus on fused deposition modeling (FDM) and fused filament fabrication (FFF) [6,7,21,22,23]. Guo et al. [12] used digital analysis to evaluate the fit of the removable partial dentures made from PEEK and manufactured by FDM. The results showed that the overall 3D deviation between the removable partial denture and the gypsum model was (0.277 ± 0.079) mm. The accuracy of the removable partial denture comprising PEEK and constructed using FDM technology can be considered clinically acceptable. A dental technique [24] for obturators using a 3D-printed PEEK framework was found to fit oral tissues well. However, there are no long-term clinical studies discussing its performance. The applications of 3D printing for the fabrication of removable PEEK prostheses and information regarding the accuracy of other additive techniques are still scarce. Thus, a more in-depth exploration of additive manufacturing methods for PEEK prostheses is needed.

## 3. Characteristics of PEEK and Its Composite Materials

### 3.1. Surface Roughness and Polishing Protocols

The surface roughness of dental materials is extremely important, as it influences microbial adhesion, biocompatibility, wear, corrosion behavior, and esthetics [25]. Reduced surface irregularities play a significant function in improving tongue sensation and in preventing dental plaque from adhering to dental prosthetics. Ra, a two-dimensional parameter, is the arithmetic mean of the absolute values of the distances from the mean line to the profile [26]. A Ra within 0.2 μm has been reported to meet the clinical requirements for prostheses according to biofilm formation. Surface roughness with a Ra of within 0.2 μm has no significant impact on plaque retention. 

The chemical components of the material, in terms of the matrix composition and fillers, play a vital role in surface roughness [27]. Previous research [28] reported that PEEK discs showed significantly higher nano-roughness than machined titanium (*p* = 0.005). For other materials, in vitro research [25] comparing PEEK and other CAD/CAM materials showed that samples of PEEK were slightly rougher than samples of polymethyl methacrylate (PMMA) and resin-based composites. The reason for this could be related to the ceramic particles added to PEEK [25,29]. To eliminate the influence of fillers, another study by Benli et al. [30] showed that pure PEEK exhibited the lowest Ra values (0.139 ± 0.017 μm) as compared to PMMA (0.192 ± 0.018 μm) after wet polishing using 2500- and 4000-grit silicon carbide paper. However, another study compared the mean surface roughness between the pure PEEK and PEEK composites, which was similar after the milling technique [8]. 

Polishing removable dental prostheses to obtain a smooth surface and to provide a superior tongue feel is important [10,31,32]. Batak et al. [8] used a contact profilometer to compare the Ra values of differently milled PEEK and its composite materials and investigated the effect of polishing on the Ra values of the polymer surfaces. The Ra results of all of the materials before and after polishing were above 0.2 μm. The unsatisfactory results may be due to noneffective polishing protocols. Therefore, proper polishing procedures should be investigated to decrease surface roughness to below 0.2 μm [8]. Sturz et al. [27] concluded that the application of 1 μm of diamond paste with a cotton buff can obtain high-gloss PEEK (Ra = 0.073 ± 0.0128 μm). Moreover, Kurahashi et al. [33] evaluated the influence of various PEEK-polishing protocols. The clinically acceptable surface roughness was obtained in all of the polishing protocols that used rubber point, “silky shine”, and “aqua blue paste”. Polishing solely using “aqua blue paste” obtained the lowest Ra values (0.009 ± 0.002 μm). Polishing protocols that used this paste resulted in more effective polishing. Similarly, Heimer et al. [34] compared four laboratory methods and three chairside polish methods and found that the use of polishing paste significantly decreased the surface roughness values. Polishing with Abraso polishing paste and Opal L polishing paste as a laboratory-side protocol yielded lower surface roughness values of 0.034 ± 0.010 μm and 0.046 ± 0.008 μm, respectively. Comparing laboratory methods, the chairside polishing protocols exhibited higher surface roughness values with the use of Prisma-gloss polishing paste (0.072 ± 0.009 μm). Importantly, a polishing paste created PEEK surfaces with lower surface roughness values. The benefit of using polishing pastes is that the combination of the paste with water leads to a fine abrasive action and a high-gloss, light-reflective surface [34]. Even if sufficient polishing is achieved on the laboratory side, the surface of a prosthesis may need to be adjusted due to malocclusion and would then need to be polished to be smooth again. Moreover, artificial aging has been proven to increase roughness values [10]. Therefore, more effective and convenient chairside polishing protocols should be designed.

### 3.2. Bacterial Plaque Retention

The characteristics of the materials employed for use in removable prostheses must be resistant to microbial colonization [35]. Chemical composition, surface-free energy, and surface roughness have the potential to affect bacterial adherence [36]. It has been demonstrated that the influence of surface roughness and surface-free energy is regarded as a dominant factor in the quality of bacterial plaque retention. Bacterial adherence on PEEK differs from that of other materials such as ceramics and metals [37,38,39].

In terms of surface roughness, Barkarmo et al. [36] compared biofilm formation on polished PEEK and blasted PEEK. They found that blasted PEEK with a rougher surface topography showed an increased number of bacteria, with the observed bacteria including *Streptococcus sanguinis, Streptococcus oralis*, and *Streptococcus gordonii*. The presence and dimensions of surface irregularities such as flaws, defects, and fractures are thought to promote bacterial attachment. Notably, an irregular surface texture is better protected against shear forces [27], which play the most decisive role in initial bacterial adhesion. 

Regarding chemical composition, the research [28] has already proven that, as compared to titanium, PEEK showed antiadhesive and antibacterial properties between 24 and 48 h against oral bacteria such as *Streptococcus oralis*. Similarly, an in vitro study by Hahnel et al. [37] evaluated multispecies biofilm on different materials (zirconia, titanium, PMMA, and PEEK) and indicated that biofilm formation on PEEK and PMMA was equal or lower as compared to zirconia and titanium. However, the PEEK material surface used in Hahnel’s study was significantly smoother than both zirconia and titanium. Another study [36] compared the surface roughness among polished PEEK, pure titanium (cp-Ti), and titanium-6 aluminium-4 vanadium (Ti6Al4V). The PEEK surfaces were significantly rougher than the cp-Ti and Ti6Al4V surfaces. The bacterial retention values for Streptococcus sanguinis were significantly higher on PEEK surfaces as compared to Ti6Al4V, which is in accordance with the study investigated by da Rocha et al. [35]. It was apparently indicated that *Streptococcus aureus* and *Candida albicans* adhesion were more likely to occur on PEEK surfaces than on titanium surfaces. As compared to PEEK, the polyetherketoneketone (PEKK) samples exhibited more bacterial adhesion [38]. Although studies focusing on bacterial adhesion to certain materials reported a high heterogeneity, PEEK was shown to facilitate the prevention of pathologies related to microbial colonization.

Materials with lower surface-free energy provide hydrophobic surfaces and are more likely to facilitate hydrophobic bacterial growth. *Candida albicans*, which are generally considered the main causative agent of denture stomatitis, are reported to be hydrophobic, and this is an important factor for the initial adhesion of *Candida albicans* [40]. An in vitro study evaluated Candida albicans biofilm formation on the surface of PEEK and titanium. The planktonic yeast cell counts in PEEK were comparable to those in titanium alloy samples; however, the sessile yeast cell counts in titanium alloys were lower than those in PEEK (*p* < 0.05) [35].

Studies evaluating the clinical bacterial adhesion of PEEK are scarce. Only one clinical report [41] used a plaque-disclosing agent to show clear plaque accumulation on both the tissue surface and polished surface of PEEK clasps after a 2-year follow-up. Thus far, no clinical complications, such as mucositis and stomatitis, have been reported in relation to PEEK removable prosthesis. However, long-term clinical trials are needed.

### 3.3. Color Stability

Pure PEEK has a brown or grayish color, which may limit its clinical application. Previous studies used titanium dioxide and ceramic particles to obtain appropriate tooth colors to mimic hard tissue, which is available in a wide range of shades, from pearl white to a wide variety of different enamel colors [42,43,44,45]. However, the colors obtained are still limited as compared to dental shade guides [44]. Therefore, PEEK can be veneered with composite resin to mimic vivid enamel shades [45,46]. Moreover, to imitate the color of the gums, the addition of ferric oxide (Fe_2_O_3_) into PEEK alters its original color to a light pink shade [47]. More research is required to provide more color options for PEEK.

Discoloration, as one form of esthetic failure, is a relevant clinical problem [48]. The discoloration of dental prostheses after long-term use may be caused by intrinsic factors and extrinsic factors [10,48]. Intrinsic factors include water absorption, the dissolution of the matrix composition or fillers, chemical reactions, and the restoration of the manufacturing mode [49]. Extrinsic factors often include penetrative dye from drink colorants such as coffee or tea, or smoking (nicotine). The adsorption of exogenous colorants onto the surface can affect color stability [48].

Regarding intrinsic factors, Liebermann et al. [50] assessed the solubility and water absorption of PMMA, composite resin, and PEEK in different aging media and for different durations. As compared to other materials, the lowest solubility and water absorption values were found in PEEK. In agreement with this statement, a clinical report [41] showed that few color and texture changes were found macroscopically in PEEK clasps after 2 years of follow-up. In terms of extrinsic factors, surface roughness and the processing of materials have a significant impact on discoloration [49]. This is because rough surfaces induce higher pigment affinity. Subjectively, the color of pressed and milled PEKK discs is very similar after polishing [51]. There is no general agreement on the effects of surface treatments and processes on color change induced by staining, and more studies are required to this end.

To simulate oral circumstances, certain studies have evaluated the color stability of PEEK after immersion in different staining media [10,52]. PEEK exhibits more color stability than denture resin materials [52]. Porojan et al. [10] investigated the long-term effects of the collective efforts of aging and different storage solutions, such as hot coffee, cold juice, and distilled water, on PEEK materials against discoloration and found that artificial aging decreased the translucency and opalescence of glazed PEEK surfaces. In addition, immersion in hot coffee demonstrated perceivable discoloration. Similarly, another study reported [52] that the highest level of discoloration was observed after immersion in a curry solution that included more orange pigments and conjugated diarylheptanoids (curcumin) [53]. Some orange and brown pigments exhibited a high affinity to the polymer phase and should be avoided while using PEEK restoration. 

It would also be interesting to perform a simulation to determine the stain removal potential. Heimer et al. [52] reported that patients should use sonic toothbrushes to remove stains from PEEK restorations. Dentists can utilize powder air abrasion to remove pigments on PEEK surfaces within the scope of professional prophylaxis. However, air abrasion may increase surface roughness values and result in the need for proper polishing, as previously recommended [27]. 

### 3.4. Wear

Wear can be described as the continuous and gradual removal of surface material or deformation resulting from mechanical interaction between two or more surfaces in contact with one another [54]. The wear of natural teeth is, to a certain degree, a physiological process, and restorative materials should not damage natural antagonistic teeth [55]. Simulations of wear loss usually focus on two-body wear interactions and three-body wear interactions. Two-body abrasion in the oral environment occurs when surfaces slide against one another without any particles being present. For example, these conditions often occur in bruxism. Three-body abrasion can be a consequence of masticatory and tooth-brushing processes. Hard particles originating from food intake or toothpaste increase the abrasive wear damage of the teeth or restorative surfaces [56].

For two-body wear abrasion, Abhay et al. [57] evaluated zirconia and PEEK crown-wear abrasion using a 3D laser scanner after 120,000 chewing cycles. For the wear of antagonistic steatite, the zirconia crowns exhibited values that were three times higher than those of PEEK crowns (*p* < 0.001). Additionally, the zirconia crowns seemed more wear-resistant than PEEK crowns (*p* < 0.001). However, this study did not include enamel as an antagonist because of standardization difficulties, and wear direction was limited to the buccolingual direction. Another study by Wimmer et al. [58] compared the two-body wear behavior of PEEK, PMMA, and nanohybrid composites (COMP). All samples, which were designed as crowns and flat specimens, simulated wear abrasion with human enamel and stainless-steel antagonists in the lateral and axial directions of force after 600,000 chewing cycles. In the lateral force application, PEEK groups exhibited significantly lower material loss than the other materials against enamel and stainless-steel antagonists. The same results were obtained in the flat groups pretested with enamel under axial load. Similarly, another in vitro study [30] compared the wear behavior of ethylene vinyl acetate (EVA), PMMA, polycarbonate (PC), PEEK, and polyethylene terephthalate (PETG) after 60,000 chewing cycles. The volume loss of PEEK was 1.084 ± 0.109 mm^3^, and the wear volume loss of the other materials was above 2 mm^3^. As mentioned above, bruxism, which can lead to temporomandibular disorders, is typical of two-body abrasion [59]. Occlusal splints are commonly prescribed for the treatment of bruxism [60]. One of the most important properties of the material used for occlusal splints is wear resistance. A clinical study [61] compared the wear behavior between acrylic resin splints and PEEK splints. The maximum depth loss and volume loss of PEEK splints were significantly less than those of acrylic resin splints. Therefore, PEEK splints had better wear resistance than acrylic resin splints. The reason as to why PEEK exhibits minimal wear loss may be related to its plastic deformation.

For three-body wear abrasion, the laboratory study by Sampaio et al. [56] compared the wear behavior of PEEK and a titanium alloy via ball-cratering tests, which is a technique to evaluate the susceptibility of components to wear volume loss under conditions simulating dental applications. In the presence of hydrated silica and distilled water slurry, the titanium alloy was more wear-resistant than PEEK. This behavior was mainly related to the lower hardness of PEEK surfaces as compared to the titanium alloy. There is little laboratory and clinical evidence concerning three-body abrasion in PEEK as compared to other materials.

### 3.5. Retention Force and Fatigue Resistance of Removable Dental Prosthesis Clasps

Although metal clasps have shown long-term stability, reliability, and high retentive capabilities, some patients have had allergic reactions to the oral mucosa. Moreover, their silver color can be esthetically unpleasing when used in removable prostheses placed on the anterior teeth. These disadvantages call for the implementation of metal-free clasp materials. Various studies have investigated PEEK as a potential replacement for metal RPD clasps. However, a standard structural design related to its properties has not been established [62].

One significant quality of clasps is their retention force, which will resist the removable prosthesis’ dislodging force during masticatory function and muscle movement [63]. When determining the fit of a clasp material to the retentive area, the clasp shape (length, width), flexibility, and undercut engagement should be considered [64]. The optimum performance of a clasp depends on the balance between these variables.

The retention force of PEEK clasps is lower than that of CoCr clasps. A finite element study [65] showed that the retention force of circumferential clasps made of PEEK ranged from 6.45 to 18.36 N, lower than that of CoCr (21.78–65.37N). This is in line with an in vitro study by Tannous [66]. The PEEK clasps provided a retention force of 1.7–8.6 N, again lower than that of CoCr (11.3–16.3N). Similarly, Micovic et al. [67] examined the retention force of three different PEEK clasps compared to cobalt–chrome–molybdenum (CoCrMo) clasps and a control group. The control group had a higher retention force than the PEEK groups (*p* < 0.001). Although there is high heterogeneity among past studies, PEEK consistently exhibited a lower retentive force than CoCr. In a clinical report, the authors manufactured PEEK circumferential clasps with an undercut of 0.25mm for patients who were diagnosed as having Kennedy Class I bilateral edentulism. In comparison to previously manufactured metallic clasps, the patients claimed a decreased amount of retentive force [18]. The required retentive force differs considerably with varying RPD designs and tooth types [68]. Frank et al. [69] suggested that a retention rate of 2.94–7.35 N was adequate in Kennedy I RPDs. However, the lowest acceptable retentive force for one clasp was determined to be approximately 1.6 N [64]. Therefore, the retentive force provided by PEEK may be clinically acceptable [63,65,66,70]. A clinical report [71] demonstrated that PEEK clasps with an undercut of 0.5 mm could provide an adequate retention force. As a result of the relatively lower retention force, PEEK clasps are recommended for patients with esthetic concerns due to the metallic appearance or who have metal allergies, and to manage periodontally compromised abutment teeth. The lower retention force benefits the maintenance of periodontal health to avoid increased bone loss. 

An essential element affecting the clinical durability of removable prosthesis clasps is fatigue resistance [63]. Clasps undergo repeated bending caused by mastication and by the insertion and removal of the removable prosthesis, which may cause fatigue failure during long-term use [68]. PEEK has a low elastic modulus (3.0–5.5 GPa), which plays an important role in fatigue testing [62]. Repeated removal and insertion of a single clasp over an analog in vitro simulated long-term clinical use. Testing clasps in this way allows for the comparison of the potential degradation of that retention force over time. It also allows for a comparison of any permanent distortion of the clasps following prolonged cyclic testing, which would be expected to have a detrimental effect on clasp retention [72]. Hussein [73] evaluated the retentive force after cyclic aging and the pull-off force of removable partial denture clasps manufactured from PEEK and GBP (graphene-based polymer) materials. The analog tooth material selected was CoCr material. Cyclic pull-off forces of 10,000 cycles and aging of 10,000 thermocyclers were applied to simulate frequent insertion and removal by the patient and the effects of the oral humid environment. The retentive force of the PEEK clasps showed statistically significantly higher values (2.248 ± 0.315 N) than the graphene-based polymer (GBP) clasps (2.018 ± 0.298 N) (*p* < 0.001). Similarly, a finite element study [64] designed PEEK clasps with undercuts of 0.25 mm or 0.50 mm. A total of 15,000 cyclic pull-off forces were performed, representing the simulated insertion and removal of the removable partial denture over 10 years, with the assumption that the patient would perform four complete cycles per day. The obtained constant-displacement fatigue test data indicated that the average load values of the PEEK clasps (mean: 2.06–3.67 N) were smaller than those of the CoCr clasps (mean: 8.26 N). This is in line with an in vitro study by Gentz et al. [70]. CoCr clasp assemblies displayed significantly higher retentive forces (11.98 N) than both thermoplastic polymers, as follows: PEKK (2.16 N, *p* < 0.001) and PEEK (2.74 N *p* < 0.001), after 15,000 insertion/removal cycles. However, after 30,000 fatigue cycles (simulating 21 years), Zheng [74] noted that the retentive force of the PEEK clasps ranged from 0.23N to 0.49N.

Different thicknesses and undercuts of clasps lead to different retention forces after fatigue cycles. Güleryüz et al. [75] evaluated the retentive force and dimensional changes in clasps with different shapes and undercuts fabricated from PEEK after 7200 mechanical cycles. The retentive force of the PEEK clasps with a thickness of 1.5 mm and an undercut of 0.50 mm had higher values (4.389–3.388 N) than those with a thickness of 1 mm and an undercut of 0.25 mm (5.459–4.141 N). Therefore, PEEK clasps were suggested to be placed in an undercut of 0.5 mm, and the cross-sectional areas of the clasps and connectors had to be thicker than those of metal clasps to provide optimal retention and strength [15,76]. This is in line with a clinical report [18]. The authors suggest the use of a deeper undercut (0.5 mm). Increasing the clasp bulkiness can provide additional retention for PEEK clasps.

## 4. Clinical Performance for Removable PEEK Prosthodontics

### 4.1. Removable Partial Dentures

Removable partial dentures (RDPs) are an inexpensive and predictable treatment option for the rehabilitation of partially edentulous patients [77]. PEEK has been introduced as a lightweight framework material. Moreover, PEEK RPDs can be fabricated via CAD/CAM techniques and conventional heat-pressing techniques (Table 1 and Table 2). CAD/CAM techniques are easily reproducible in the event of failure [78], and PEEK frameworks can be easily relined in the case of resorption [64]. Moreover, PEEK RPDs produced by rapid prototyping exhibit good results in terms of framework accuracy [13]. 

Various clinical reports [18,71,78] have shown that PEEK (Bio-HPP) RPDs have a lower specific weight than CoCr RPDs, providing high patient satisfaction and comfort [18]. A randomized controlled trial [83] compared the satisfaction of 10 patients asked to wear metal and PEEK framework RPDs for 3 months. Patients were more satisfied with the milled PEEK framework RPDs than with the CoCr framework RPDs. However, another randomized controlled crossover pilot trial [82] investigated the satisfaction of 26 patients after 4 weeks, 6 months, and 12 months of wearing CoCr and PEEK frameworks. PEEK frameworks did not have a significant impact on Oral Health Impact Profile scores. Therefore, further studies detailing long-term clinical performance are necessary.

In terms of biological characteristics, a finite element analysis compared the stress distribution and displacement on tissues (abutment periodontal ligament, bone, mucous membrane) and under masticatory forces among PEEK, titanium alloy, and CoCr RPDs frameworks [84]. The maximum von Mises stress of periodontal ligaments in the PEEK group was lower than both the titanium alloy and the CoCr alloy, indicating that PEEK was more conducive to protecting the PDL of the abutment. Therefore, PEEK RPDs may benefit periodontally compromised abutments. Regarding the mucosa stress, the maximum stress values of the frameworks fabricated with PEEK were significantly higher than those of the titanium alloy and the CoCr alloy (*p* < 0.05). Stress that is not equally distributed by the mucosa and other pertinent tissues could cause localized pain or even excessive absorption of the alveolar bone. A clinical study [79] compared yearly dimensional changes in the edentulous residual ridges of patients wearing milled PEEK RPDs and those who were not via intraoral scans. No statistically significant difference was seen regarding the mean 3D distance at the corresponding points of the denture-bearing areas (represented by the changes in the height of the edentulous ridge) during follow-up (*p* = 0.506). In contrast, another clinical trial radiographically evaluated the effects of the two different CAM fabrications (milled and pressed) of PEEK frameworks on the distal extension portion of the ridge and concluded that both groups showed bone loss [80]. The inconsistent standards, testing procedures, and findings make it challenging to compare these two studies. In the same study [80], pressed BioHpp exhibited lower bone loss around the abutments than milled BioHpp (*p* = 0.0001). Conversely, regarding the distal extension portion of the ridge, pressed BioHpp exhibited higher bone loss than milled BioHpp (*p* = 0.003). According to the authors, this effect may be due to the improved retention of milled BioHpp compared to pressed BioHpp, which was observed throughout the study’s follow-up period. Long-term and large-scale clinical studies are necessary to assess the prevalence of this effect because similar changes were not reported in other studies.

Compared to metal alloys, some studies report higher patient satisfaction when using PEEK clasps regarding satisfaction with aesthetics and appearance [81,85]. The retention force of RPDs is mainly provided by the clasps engaging undercuts around the healthy abutment. The clasps have a horizontal force exerted on the abutment teeth over multiple insertion/removal cycles. Such bad stress leads to periodontal affection and successive alveolar bone resorption, especially to the abutment teeth neighboring the defect [11]. As a result of the flexibility of PEEK, it may be beneficial for abutment teeth. A clinical report [41] on PEEK clasps showed that the abutment teeth exhibited no abnormal mobility, and the gingiva around the abutment teeth exhibited no inflammation. Moreover, PEEK clasps are gentler on the enamel and restorative ceramic materials than conventional CoCr clasps [71]. However, more long-term clinical evidence is required.

### 4.2. Double Crown-Retained RPDs

Double crowns are successfully used as retainers for removable partial dentures and consist of a primary coping crown and a precisely fitted secondary crown [86]. Gold alloys, CoCr, and titanium are examples of metal alloys used in telescopic crown systems, which are typically manufactured via the conventional heat-pressing technique. High success with such designs, especially in the long term, has been reported [87]. CAD/CAM fabrication techniques have led to the use of PEEK in the construction of telescopic crowns. 

A clinical report [88] described the manufacture of a double-crown-retained prosthesis with a PEEK framework that resulted in the functional and esthetic rehabilitation of the patient. However, high costs may restrict its application. A randomized clinical trial [86] investigated the retention forces of the CAD/CAM-milled PEKK and PEEK double-crown-supported removable partial dentures using a digital force gauge. After a year of clinical use, double-crown-retained RPDs made from PEEK and PEKK in combination with primary copings made from ZrO_2_ demonstrated a significant increase in the dislodging force. Additionally, all patients were satisfied with the retention and esthetics of their PEKK and PEEK double-crown-retained RPDs. There is a need for future prospective long-term clinical trials with larger patient samples due to the limited available scientific evidence obtained.

### 4.3. Overdentures

Overdentures are an alternative choice for edentulism. Compared to conventional dentures, overdentures can provide more stability, especially in mandibular dentures [89]. Overdentures can be divided into tooth-supported overdentures and implant-supported overdentures. On the one hand, some clinical studies have used PEEK as a framework for overdentures. For implant-supported overdentures, Kortam et al. [90] investigated the clinical and radiographic outcomes of CoCr and PEEK frameworks after a 1-year follow-up period among eight patients. The survival rate showed significant differences (Metal: 83%, PEEK: 100%). The three implants in the same patients failed in the first 6 months. The author thought this may be attributed to more forces being transmitted to the implants in the metal group. However, large-scale clinical trials must be conducted. For tooth-supported overdentures, a clinical report [91] designed a PEEK framework and a metal post/coping/ball attachment and obtained good clinical performance. 

On the other hand, some clinical studies have used PEEK to help retain implant-supported overdentures. Sharaf et al. [92] designed novel PEEK-retentive elements in mandibular overdentures and measured the retention and patient satisfaction compared to metal housings and nylon-retentive elements. The author stated that PEEK provided more retention than conventional materials and that it led to improved patient satisfaction. Similarly, another clinical trial [93] evaluated clinical, prosthetic, and patient-based outcomes of a milled bar with PEEK and metal housings for inclined implants supporting mandibular overdentures. The metal group showed a significantly higher plaque score and marginal bone resorption compared to the PEEK group after a year-long follow-up. Additionally, the PEEK group reported higher satisfaction with retention, stability, speech, and esthetics. PEEK housing a milled bar may be a successful alternative to the conventional metal housing. Furthermore, Mangano et al. [94] rehabilitated 15 patients with maxillary overdentures supported by PEEK bars, and a high accuracy and an ideal passive fit were obtained.

### 4.4. Obturator Prosthesis

Maxillectomy defects may be due to trauma, pathological conditions, or the surgical resection of oral tumors [95]. The subsequent principal issue is oronasal communication, leading to impairment in masticatory function, swallowing, speech intelligibility, and facial esthetics. The primary point of prosthetic rehabilitation is to eliminate oronasal communication [95]. Maxillary obturators, which consist of an obturator bulb and a denture component, are mainly constructed for the rehabilitation of maxillary defects, providing durable and excellent retention, stability, and support. A major issue with conventional maxillary obturator prostheses pertains to their weight [96]. The larger the defect, the heavier the prosthesis. Ding et al. [24] proposed a 3D-printed PEEK framework obturator with a precise fit, excellent retention, and reduced weight. PEEKs light weight enables it to be widely used in maxillofacial prostheses (Table 3). 

A randomized controlled trial [85] investigated patient satisfaction with conventional clasp-retained obturators and CoCr and PEEK attachment-retained maxillary obturators. A noticeable grey-colored clasp in the conventional group was unpopular with the patients. The PEEK group was esthetically superior. In terms of retention and stability, PEEK and metal attached-retained groups felt comfortable during speech due to the accurate fit and simpler insertion path as compared to the conventional group. However, another technique [97] introduced a fully digital workflow for milled PEEK obturators. The author suggests that the esthetics of PEEK and polylactic acid obturators are too poor for anterior use and that this may be improved by coating a PEEK obturator prosthesis with resin or by 3D printing a multicolored polylactic acid obturator prosthesis. 

As regards bone loss, Sharaf et al. [85] showed that PEEK and metal groups exhibited lower mean bone loss than groups with conventional clasp-retained obturators. This is in line with a finite element study [98]. This study simulated an implant-supported palatal obturator prosthesis made of the following three different materials: PEEK, titanium, and Co-Cr alloys. Although the PEEK frameworks were prone to an increased risk of the prosthetic screws loosening and even fracturing, as compared to the metallic alloys, they can be used in frameworks due to their ability to decrease bone strain around the implants and the bar structure.

**Table 3:** Clinical studies on obturator prostheses.

Author, Year	Type of the Study	Participants	Intervention	Follow-up	Results	Conclusions
Sharaf et al. [85]	Random control study	*N* = 18 Females: 6 Males: 12	PEEK/metal attachment-retained obturators and conventional clasp-retained obturators	1 week and 3, 6, 9, and 12 months	**Obturator functional scale scores:** PEEK and metal group scores decreased for looks, talking, and prosthesis insertion **Bone loss:** PEEK and metal groups showed lower values than conventional clasp-retained obturators	Regarding esthetics and appearance satisfaction, PEEK can be used in obturators
Ding et al. [24]	Technique	Not reported	Maxillary obturator with a 3D-printed PEEK	Not reported	Precise fit, excellent retention, and reduced weight of PEEK framework compared to metal framework	
Guo et al. [97]	Technique	Not reported	Maxillary obturator with a milled PEEK	Not reported	A fully digital workflow for the design and manufacture of PEEK obturator was shown	
Tasopoulos et al. [99]	Case report	Females: 47	Two-piece hollow bulb maxillary obturator prosthesis	1 year	The resilience and long-term functionality of the PEEK clasps and the biological and structural stability of the two-piece obturator parts have yet to be determined	
Costa-Palau et al. [96]	Case report	Females: 58	Maxillary obturator with a milled PEEK	6 months	The strength and appearance of the PEEK and acrylic resin were satisfactory	

## 5. Conclusions

PEEK is an innovative material that has attracted great interest for use in removable prostheses, such as RPDs (clasp and framework), double crown-retained RPDs, implant-supported overdentures, and obturator prostheses. As regards the limitations of this review, no meta-analysis, including clinical and laboratory studies, was performed for inadequate evidence. On the basis of the findings of the articles reviewed, the following conclusions can be drawn:

1. The accuracy of the conventional lost-wax technique and subtractive techniques is adequate for the manufacture of removable prostheses. However, to fabricate removable prostheses with complex designs, addictive manufacturing must be explored, and the accuracy of such manufacturing methods must be investigated;
2. The surface roughness of PEEK is clinically accepted, and the use of pastes represents an effective method for PEEK polishing. However, as compared to laboratory polishing protocols, more convenient chairside polishing protocols for PEEK should be investigated;
3. PEEK with lower surface-free energy provides hydrophobic surfaces and is more likely to facilitate the growth of hydrophobic microorganisms, such as *Candida albicans*. In terms of high heterogeneity, laboratory studies need to be more standardized. Only one report revealed the plaque accumulation of PEEK and the methodological quality of the studies that need to be addressed in future clinical studies;
4. PEEK is available in a wide range of shades, from pearl white to pink shades. On the basis of the lower solubility and water absorption values, PEEK may have higher color stability compared to PMMA and composite resin. It should be noted that some orange and brown pigments show a high affinity for the polymer phase and should be avoided while using PEEK restoration. However, only one clinical study reported fewer colors and textures in PEEK clasps. Long-term and large-scale clinical trials should be investigated;
5. As a result of plastic deformation, PEEK appears to be more wear-resistant. The three-body abrasion evidence is limited, especially in studies that compare PEEK with other materials;
6. The retention of PEEK clasps is clinically accepted based on existing evidence. As compared to metal clasps, a deeper undercut (0.5 mm) and increased bulkiness can provide additional retention for PEEK clasps. Future research should focus on its clinical survival rates considering the complexity of the oral environment, as it represents a promising new platform for investigation;
7. After summarizing various clinical reports, techniques, and short-term clinical trials, we have concluded that PEEKs clinical performance in removable dental prostheses is satisfactory and promising. However, large-scale and long-term clinical controlled trials should be conducted.

## Figures and Tables

**Table 1:** General characteristics of clinical studies on removable partial dentures. PEEK: polyetheretherketone; CoCr: cobalt-chromium.

Author, Year	Type of the Study	Participants	Age (Mean/Range)	Intervention	Follow-up	Dropouts
Lo Russo et al., 2022 [79]	Case–control study	*N* = 10	46–72	Milled PEEK framework	12.5, 13 months	Not stated
		Males:3				
		Females:7				
Lo Russo et al., 2021 [17]	Technique	Not reported	Not reported	Milled PEEK framework	6 months	Not stated
Elsarrif et al., 2021 [80]	Random control trial	*N* = 14	35–50	Group I: Milled PEEK framework, Group II: Pressed PEEK framework	6, 12 months	Not stated
		Males:				
		Not reported				
		Females:				
		Not reported				
Nishiyama et al., 2020 [81]	Technique	*N* = 2	68,67	PEEK clasps	6 months	Not stated
		Males:				
		Not reported				
		Females:				
		Not reported				
Ali et al., 2020 [82]	Random control trial crossovers	*N* = 26	39–85	CoCr frameworks, Milled PEEK frameworks	4 weeks, 6,12 months	7
		Males: 11				
		Females: 15				
Mohamed et al., 2019 [83]	Clinical trial	*N* = 10	30–50	CoCr frameworks, Milled PEEK frameworks	3 months	Not stated
		Females:				
		Not reported				
		Males:				
		Not reported				
Ichikawa et al., 2018 [41]	Case report	Female	84	Milled PEEK clasps	2 years	Not stated
Harb et al., 2018 [18]	Case report	Female	56	Milled PEEK frameworks	Not reported	Not stated

**Table 2:** Clinical outcome of clinical studies on removable partial dentures. PEEK: polyetheretherketone; CoCr: cobalt-chromium; MDSQ: McGill Denture Satisfaction Questionnaire.

Author, Year	Biological Outcome	Patient Satisfaction	Prosthodontic Outcomes
Lo Russo et al., 2022 [79]	Vertical height and 3D changes of residual ridges are not significantly different with or without PEEK RPDs.	Not reported	Not reported
Lo Russo et al., 2021 [17]	Not reported	Not reported	No tooth or base debonding or other clinical complications
Elsarrif et al., 2021 [80]	Milled BioHpp frameworks had a greater effect on bone resorption around the abutments than pressed BioHpp while at the residual ridge area, pressed BioHpp showed more bone height changes than the milled one.	Not reported	Not reported
Nishiyama et al., 2020 [81]	Not reported	Improved patient satisfaction scores	No clinical complications.
Ali et al., 2020 [82]	No significant differences were found in periodontal indexes.	Significant improvements to MDSQ in both groups	Not reported
Mohamed et al., 2019 [83]	Not reported	Increased patient satisfaction of PEEK RPDs as compared with the conventional metal framework material	Not reported
Ichikawa et al., 2018 [41]	No particular occlusal or periodontal problems	Satisfaction	Few color and texture changes in PEEK and no other clinical complications
Harb et al., 2018 [18]	Not reported	Satisfaction	Not reported

## Data Availability

Not applicable.
